# Supplementary material for: Triamcinolone acetonide-loaded chitosan-polyethylene glycol hydrogel for preventing esophageal stricture post-endoscopic submucosal dissection
Source: Regen Biomater. 2026 Jan 14;13:rbag002. doi: 10.1093/rb/rbag002 (PMC12915572; doi:10.1093/rb/rbag002)
Supplement: rbag002_Supplementary_Data [file rbag002_supplementary_data.docx]

**Triamcinolone acetonide-loaded chitosan-polyethylene glycol hydrogel for preventing esophageal stricture post endoscopic submucosal dissection**

Yaqiang Li^1, 2^^†^, Rui Gao^3†^, Zhen He^1^, Kuiliang Liu^1^, Zujian Feng^4^, Pingsheng Huang^3^, Chuangnian Zhang^3^, Haijun Hou^5^, Baohong Xu^2^, Jianduo An^2^, Yan Zhao^1^*, Weiwei Wang^4^*, Shutian Zhang^1^* and Peng Li^1^*

1. Department of Gastroenterology, Beijing Friendship Hospital, Capital Medical University, State Key Laboratory of Digestive Health, National Clinical Research Center for Digestive Disease, Beijing Key Laboratory of Early Gastrointestinal Cancer Medicine and Medical Devices, Beijing 100050, P. R. China.

2. Department of Gastroenterology, Beijing Luhe Hospital, Capital Medical University, Beijing 101100, P. R. China.

3. State Key Laboratory of Advanced Medical Materials and Devices, Institute of Biomedical Engineering, Chinese Academy of Medical Sciences and Peking Union Medical College, Tianjin 300192, P. R. China.

4. College of Life Sciences, Key Laboratory of Bioactive Materials (Ministry of Education), State Key Laboratory of Medicinal Chemical Biology, Nankai University, Tianjin 300071, P. R. China.

5. Department of Pain Medicine, Guanganmen Hospital, China Academy of Chinese Medical Sciences, Beijing 100053, P. R. China.

† These authors contributed equally to this work.

Correspondence address. E-mail:

yanzhao@mail.ccmu.edu.cn (Y.Z.);

wwwangtj@163.com, wangweiwei@nankai.edu.cn (W.W.);

zhangshutian@ccmu.edu.cn (S.Z.);

lipeng@ccmu.edu.cn (P.L.)

**Abstract**

Postoperative esophageal stricture remains a significant challenge following endoscopic submucosal dissection (ESD), with limited effective prophylactic options in clinic. Here, we report the development of an injectable and tissue-adhesive hydrogel drug delivery system composed of quaternary ammonium chitosan and polyethylene glycol (QCS-PEG), which was loaded with triamcinolone acetonide (QCS-PEG@TA), designed to mitigate post-ESD strictures. *In vitro* assays demonstrated that the hydrogel formulation modulated macrophage polarization and inhibited fibroblast migration. In an ESD-induced porcine esophageal stricture model, treatment with drug-encapsulated hydrogel efficiently suppressed esophageal stricture and promoted tissue repair, which were superior over drug or hydrogel alone. Histological and immunohistochemical analyses revealed that the administration of hydrogel formulation reduced fibrosis and inflammatory cell infiltration in esophageal tissues. These findings suggest that QCS-PEG@TA hydrogel provides mechanical support, inflammation-modulatory and pro-healing effects that collectively prevent stricture formation, offering a clinically translatable approach to improve therapeutic outcomes after ESD.


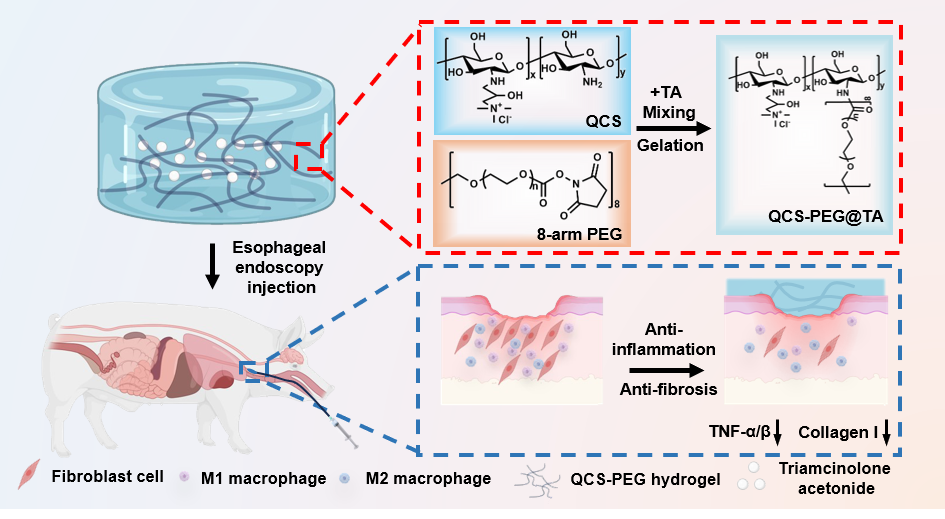


**Graphic abstract**

**Keywords**: endoscopic submucosal dissection, chitosan hydrogel, esophageal stricture, triamcinolone acetonide, tissue repair

**Introduction**

Endoscopic submucosal dissection (ESD) has emerged as the gold-standard treatment for early esophageal cancer, enabling precise resection of neoplastic mucosa, while preserving the integrity of surrounding healthy tissue [1-3]. This minimally invasive method not only avoids extensive esophagectomy, but also provides good oncological outcomes and a high cure resection rate [4]. However, when the mucosal defect involves more than 75% of the esophageal circumference, postoperative stricture formation becomes a frequent complication, with incidence rates reaching 70–90% [5, 6]. Resulting esophageal narrowing can lead to severe dysphagia, nutritional compromise, and substantial long-term morbidity [7-9].

The core pathogenesis of postoperative stricture involves excessive extracellular matrix accumulation, predominantly type I and III collagen. This process is driven by macrophage activation and subsequent cytokine release, which promote the differentiation of both resident fibroblasts and transdifferentiated epithelial cells into myofibroblasts [10-15]. The accompanying long-term inflammation is characterized by increased expression of inflammatory factors such as TNF-α, which maintains fibrosis by maintaining NF-κB mediated pro-fibrotic signaling [16, 17]. Current clinical therapeutic strategies for post-ESD esophageal stricture, including local corticosteroid (triamcinolone acetonide, TA) injection and serial balloon dilation, have demonstrated limited efficacy [18-21]. Local hormone injection aims to suppress the inflammatory response, but often requires repeated administrations, which may increase the risk of systemic side effects [22]. Balloon dilation, although widely used, provides only temporary relief and is frequently associated with complications such as esophageal perforation, bleeding, and restenosis [9, 23]. These limitations highlight the urgent need for innovative, long-lasting interventions that can effectively prevent stricture formation and improve patient outcomes.

Emerging therapeutic strategies, such as mesenchymal stem cell (MSC) therapy and biodegradable scaffolds, have been investigated as potential solutions to mitigate post-ESD stricture [20, 24, 25]. While cell therapy offers potential anti-inflammatory and regenerative benefits, its high cost and logistical complexity limit widespread clinical adoption. Similarly, biological scaffolds, despite their mechanical support, often face issues of displacement, detachment, and even perforation in the esophageal environment, severely restricting their clinical utility. This highlights the critical need for a multifunctional scaffold capable of combining mechanical support with robust anti-inflammatory and anti-fibrotic effects to effectively target the underlying mechanisms of stricture formation. Hydrogels have recently garnered attention as promising biomaterials due to good biocompatibility, tunable mechanical properties, and capacity to inhibit inflammation and delay fibrosis [25-33]. However, their application in preventing esophageal stricture has not been demonstrated.

In this study, we developed an injectable QCS-PEG hydrogel incorporating triamcinolone acetonide (QCS-PEG@TA), designed to provide mechanical support and localized anti-inflammatory effects at post-ESD wound sites. We characterized the hydrogel’s physicochemical properties, tissue adhesiveness, and drug release potential, and evaluated its biological performance *in vitro*, including macrophage polarization and inhibition of fibroblast migration.  Using a porcine ESD model, we further demonstrated that QCS-PEG@TA significantly reduced inflammatory responses, collagen deposition, and stricture formation. These findings establish QCS-PEG@TA as a multifunctional, clinically translatable strategy for preventing esophageal strictures following ESD.


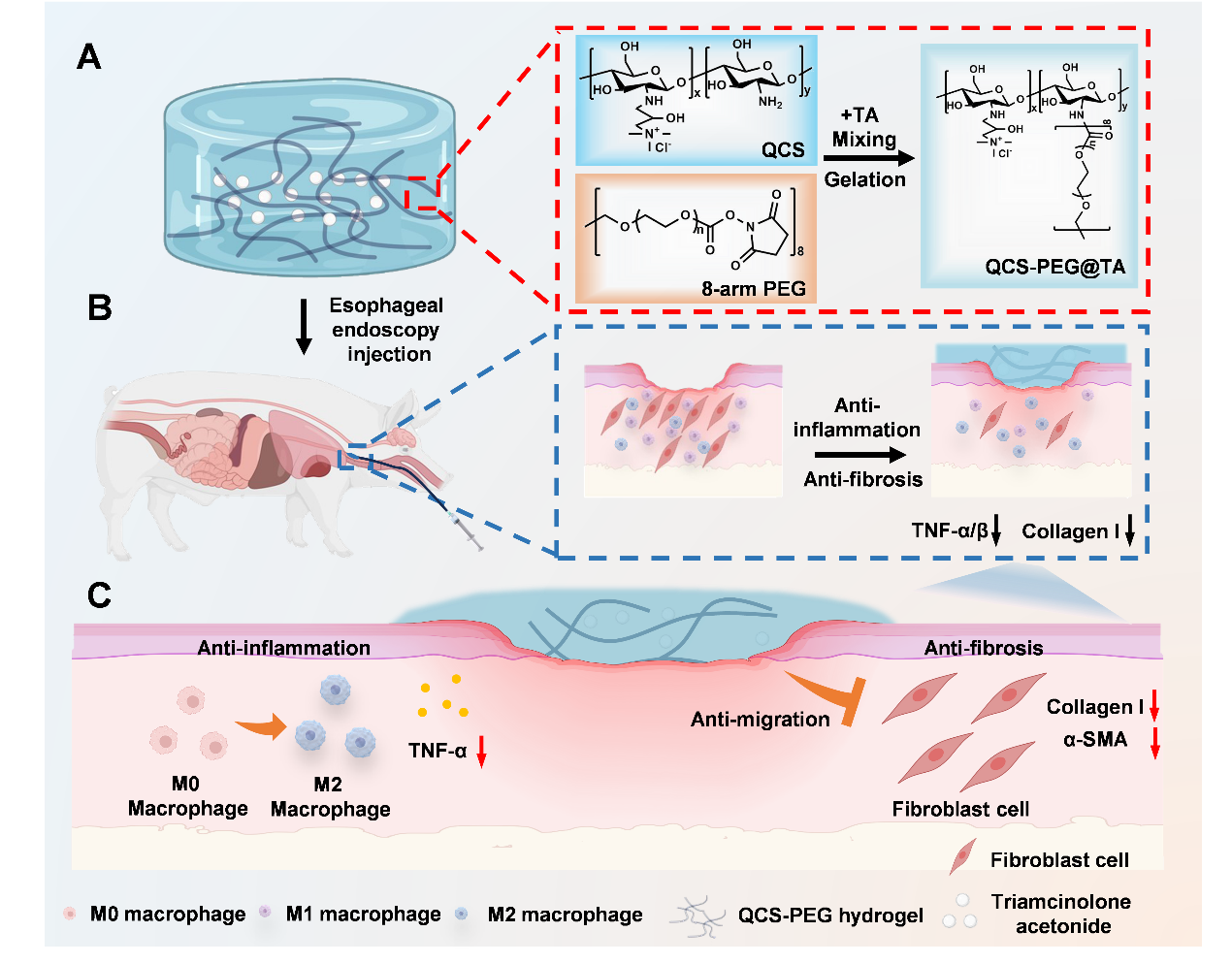


**Figure 1. Schematic diagram of QCS-PEG@TA hydrogel preventing stricture after ESD operation.** (A) The hydrogel is synthesized via gelation of quaternized chitosan (QCS) and 8-arm polyethylene glycol (8-arm PEG) with the addition of triamcinolone acetonide (TA), as depicted by molecular diagrams. (B) Upon endoscopic injection into the submucosal layer of a porcine esophagus, the hydrogel forms a biocompatible barrier, releasing therapeutic agents locally. (C) Anti-inflammatory effects are demonstrated by increased M2 macrophage activity and reduced TNF-α/β levels, while anti-fibrotic action is evidenced by decreased collagen I deposition and α-SMA secretion. Such a dual-pathway regulation addresses post-ESD stenosis, providing a translational approach for the treatment and management of esophageal stricture in clinical settings.

**Materials and Methods**

**Materials**

Chitosan (CS, Mw = 150-400 kDa, 85% deacetylation) was obtained from Shandong Haidebei Biotechnology Co., Ltd. (Shandong, China). Triamcinolone acetonide injection (40 mg/mL) was purchased from Kunming Jida Pharmaceutical Co., Ltd. Eight-arm PEG active ester (Mw = 40 kDa, purity = 95%) was obtained from Xiamen Sinopeg BIOTECH Co., Ltd. FITC-labeled anti-CD86 antibodies (Biolegend, 105006), PE-labeled F4/80 antibodies (Biolegend, 123110) and APC-labeled CD206 (Biolegend, 141708) were purchased from BioLegend (San Diego, California, USA). Cell counting kit-8 assay kit (CCK-8) was purchased from Solarbio (Beijing, China). Live/dead assay was provided by Beyotime Biotechnology (Beijing, China). TNF-α (CSB-E04741m) and TGF-β (CSB-EL023453MO) ELISA kit were purchased from CUSABIO (Wuhan, China).

**Preparation of QCS-PEG@TA hydrogel**

For synthesis of QCS-PEG@TA hydrogel, two solutions were prepared. Solution A containing varying concentrations of QCS (0.5%, 1%, 2%, and 3%) were prepared by dissolving the chitosan in 10 mL of ultra-pure water and subjecting it to ultrasound treatment in a water bath at 50℃ for 2 hours. Solution B was prepared by mixing 0.5 g 8-arm PEG in 2.5 mL triamcinolone suspension (40 mg/mL) and 7.5 mL of ultra-pure water under stirring at 200 rpm for 30 minutes. When preparing QCS-PEG hydrogel, 0.5 g 8-arm PEG was dissolved with 10 mL of ultrapure water to observe solution B. Solution A and B were then mixed in a 5:3 volume ratio to get the QCS-PEG@TA hydrogel. Scanning electron microscopy (SEM, MIRA LMS; Tescan, Czech Republic) was used to investigate the morphology of the hydrogels at an acceleration voltage of 3 kV.

**The injectability of the hydrogel**

Solutions A containing varying concentrations of QCS (0.5%, 1%, 2%, and 3%) and solution B containing 5% 8-arm PEG were combined rapidly. Then, the mixed solution was quickly drawn into the syringe and injected. The small bottle flipping method was used to determine the gel state.

**Injection thrust experiment**

The injection performance was tested using an automatic digital force gauge (HBO, Wenzhou Haibao Instrument Co., Ltd.). After mixing solutions A and B, the hydrogel was extruded immediately at a rate of 0.5 mL/s in a 10 mL syringe containing an endoscope catheter at 37℃. The maximum thrust value was recorded.

**The rheological properties of the QCS-PEG@TA hydrogel**

The rheological behavior of the hydrogels was analyzed using a certain brand of modular compact rheometer (Anton Paar, MCR 302, Austria). The elastic modulus (G’) and viscosity modulus (G’’) were measured in a frequency range of 1 – 100 rad s^−1^ with a strain of 1%, as well as a strain range of 1 – 100% with the frequency of 1 rad s^−1^ at 37℃, respectively. The G’ and G’’ as a function of time were measured at a fixed angular frequency of 1 rps and a strain of 1%.

**The tensile test**

QCS-PEG@TA and QCS-PEG hydrogel were uniformly applied to surfaces such as plastic, metal, glass, silicone, esophageal inner wall tissue, and wood, with a coating area of 10 mm × 10 mm. The tensile test was conducted using a universal tensile machine (Instron, USA) at a speed of 10 mm/min. The test was terminated upon complete separation of the substrates, and shear strength was quantified for each sample. The cyclic compression test was conducted also using a universal tensile machine (Instron, USA). The hydrogel was cyclically compressed at a rate of 10 mm/min to 20% of the strain (2 mm) for 5 cycles.

**Degradation test**

Following the even mixing of solutions A and B, the solidified hydrogel was immersed in phosphate-buffered saline (PBS) at 37℃. After freeze-drying, the hydrogel’s weight was measured at 24-hour intervals to monitor mass loss over time.

**Drug release test**

First, 2 g hydrogel containing 20 mg TA was added to 25 mL PBS solution containing 1% Tween 80, and was shaken at 90 rpm in a shaker at 37℃. Then at each time point, 5 mL of release solution was taken out and placed in a 4℃ refrigerator for testing, and 5 mL of fresh buffer solution was added. Then, an ultra-micro spectrophotometer (N60, NanoPhotometer) was used to detect the absorption peak of TA at 254 nm wavelength.

**Cytotoxicity evaluation of QCS-PEG@TA hydrogel**

First, the hydrogel and DMEM medium were mixed at a volume ratio of 1:5, and then placed at 37℃ for 48 hours to obtain the extract. Then, the cytotoxicity of QCS-PEG@TA hydrogels was evaluated by live/dead staining. Briefly, L929 cells were seeded in 12-well plates at a density of 20 000 cells per well and incubated at 37℃ in an incubator for 24 h under humidified conditions of 5% CO_2_. Further, the extract of QCS-PEG@TA hydrogel was added to the culture medium in the form of liquid exchange. After incubating for 1, 2, and 3 days, the medium was discarded, and 250 μL of a detection working solution containing 1 μL of calcein AM and 1 μL of PI was added into each well according to the manufacturer’s steps. After incubating in a dark environment at 37 ℃ for 30 minutes, an upright microscope (DMi8 manual, Leica, Germany) was used to observe the cell state.

The activity of fibroblasts was validated using Cell Counting Kit-8 (CCK-8) assay. In short, L929 fibroblasts were seeded into a 96-well plate at a density of 2000 cells per well and cultured in a standard culture environment at 37℃ and 5% CO_2_. After 24 hours, the extract diluted to 20% with DMEM medium was added to each well, and the medium without the extract was used as a blank control. The culture medium containing the extract was changed every day. After culturing for 1, 2, or 3 days, the culture medium in each well was replaced with 200 μL DMEM medium containing 10 μL CCK-8 solution. Then, the cells were incubated for 1 h and measured using a microplate reader (3001, Thermo Fisher Scientific) at a wavelength of 450 nm.

**Macrophage polarization**

Bone marrow-derived macrophages (BMDMs) were isolated from C57BL/6 mice (6 weeks old, Vital River Laboratory, China) using the previous method [34]. After lysing red blood cells, the collected cells were seeded in 6-well plates and cultured with RPMI 1640 medium supplemented with 10% heat-inactivated fetal bovine serum and 20 ng mL^−1^ M-CSF (MCE, HY-P7085). After incubation for 6 days, adhered BMDMs were cocultured with IL-4 (40 ng mL^−1^, PeproTech, 214-14) or hydrogels for 2 days. Then, BMDMs were stained with PE-labeled F4/80 antibodies (Biolegend, 123110) and APC-labeled CD206 (Biolegend, 141708) and analyzed by flow cytometry (C6, BD, USA). The levels of TNF-α and TGF-β in the cell supernatant were detected by ELISA method (TNF-α: CSB-E04741m, CUSABIO; TGF-β: CSB-EL023453MO, CUSABIO).

***In vitro* assessment of cell migration**

For cell migration assay, L929 cells were seeded in a 12-well plate at a density of 20 000 cells per well. Further, the extract of QCS-PEG@TA hydrogel was added to the culture medium in the form of liquid exchange. After incubating for 0 and 2 days, the medium was discarded, and 250 μL of a detection working solution containing 1 μL of calcein AM was added into each well according to the manufacturer’s steps. After incubating in a dark environment at 37℃ for 30 minutes, an upright microscope (DMi8 manual, Leica, Germany) was used to observe the cell state. ImageJ software was used to measure cell migration ratio.

**Pig ESD model**

All animal experiments were approved by Animal Experiment Ethics Committee of Beijing Friendship Hospital (24-7001, Capital Medical University). Bama Miniature pigs were purchased from Beijing Farm Animal Research Center Chinese Academy of Sciences (Beijing, China), weighing between 25 and 30 kg. Twelve miniature pigs were assigned into Control, TA, QCS-PEG, and QCS-PEG@TA groups (n = 3), using a random digit’s table. All animals received a 48-h half-liquid diet, and fasted for 24 h before the procedure.

Twelve miniature pigs were selected, fasted for 72 hours, and deprived of water for 12 hours prior to surgery to ensure optimal preoperative conditions. Anesthesia was induced with intramuscular ketamine hydrochloride (1 mg/kg), followed by endotracheal intubation and maintenance with isoflurane. Vital signs, including respiratory rate, heart rate, and oxygen saturation, were continuously monitored throughout the procedure to ensure anesthetic depth and physiological stability. The ESD procedure was performed by a skilled endoscopist. Following a submucosal injection of methylene blue and saline, a gastroscope (GIF-Q260J, Olympus, Tokyo, Japan) with a transparent cap was used to visualize the esophagus, and a 5-cm mucosal segment was carefully resected. Hot coagulation forceps (FD-410LR, Olympus, Tokyo, Japan) were used for ensuring hemostasis.

Both groups of animals underwent full circumferential esophageal ESD, followed by immediate application of hydrogel. The QCS-PEG and QCS-PEG@TA groups evenly sprayed 8 mL of hydrogels onto the wound surface using a modified spray tube, while the hormone local injection group injected 1 mL (40 mg) of triamcinolone acetonide at multiple points under the residual mucosa of the local wound surface. No additional procedures were performed in the control group.

**Histological analysis**

Following the macroscopic evaluation, the esophageal specimens were fixed in a 10% formalin buffered solution, embedded in paraffin, processed into sections, and then stained with hematoxylin-eosin (H&E) and Masson’s trichrome staining according to the manufacturer’s steps.

**Immunohistochemical staining**

After rehydration and membrane rupture, the slices were treated with citrate antigen retrieval solution (Cat No. ZLI-9065, zsbio). Then the slices were sealed with goat serum at 37℃ for 30 minutes. Afterwards, the slices were incubated with IL-1β (Cat No. AF7209, Beyotime), TNF-α (Cat No. bs-2150R, Bioss), TNF-β (Cat No. bs-0093R, Bioss), Cytokeratin 5 (Cat No. bs-1060R, Bioss), CD31 (Cat No. bs-0195R, Bioss), Collagen I (Cat No. bs-0578R, Bioss), α-SMA (Cat No. bsm-33187M, Bioss) overnight at 4℃, respectively. On the second day, the slices were washed and incubated with HRP-conjugated Goat anti-Rabbit IgG (H+L) (Cat No. PV-6000D, zsbio) at 37℃ for 1 h, followed by staining with DAB staining solution (Cat No. PV-6000D, zsbio). Finally, the slices were stained with hematoxylin and dehydrated before being sealed. Then, an upright fluorescence microscope (BX53F, OLYMPUS) was used to capture fluorescent sections.

**Statistical Analysis**

Statistical analysis was performed using Graph-Pad Prism 8 (GraphPad Software) and data were expressed as mean ± SD. Comparisons between two groups were performed with unpaired Student’s t-test, while for multiple group comparison, one-way ANOVA was used with Bonferroni post correction. Statistical significance is denoted by **P* < 0.05, ***P* < 0.01, and ****P* < 0.001. ns: no significance.

**Results**

**Preparation and characterization of QCS-PEG@TA hydrogel**

An injectable hydrogel (QCS-PEG) was fabricated by crosslinking QCS with 8-arm PEG to form amide bonds. Upon incorporation of TA, the resulting formulation was designated as QCS-PEG@TA. Gelation time was modulated by adjusting the QCS concentration. Rapid gelation was observed at 3% QCS (6.3  ±  1.5  s), but this led to a high risk of catheter blockage during injection. Conversely, lower concentrations (1% and 0.5%) resulted in prolonged gelation (351.7  ±  40.3  s and 1213.7  ±  80.8  s, respectively), which may compromise luminal retention. A concentration of 2% QCS enabled consistent gel formation within 72.3  ±  8.3  s, providing optimal balance between injectability and in situ usability **(Figure 2A, B)**. The gel formation evaluated by the oscillating time scanning test (angular frequency of 1 rad/s with strain of 1%) showed that the hydrogel turned into a solid-dominated behavior (G’ > G’’) within 76 seconds (Figure S1A). The squeezing force of the hydrogel was 23.44 ± 1.47 N, while that of the saline control group was 2.2 ± 0.19 N (Figure S1B). The force was within the clinically acceptable range of manual endoscopic injection. The hydrogel exhibited uniform extrusion without needle clogging, and resistance during injection remained manageable throughout the procedure.

To evaluate their mechanical stability and viscoelastic properties under varying conditions, oscillatory scanning experiments were conducted to assess strain- and frequency-dependent responses **(Figure 2C, D)**. Frequency sweeps (0.1 – 100 rps) revealed that G’ consistently exceeded G’’ across the entire range, indicating a predominantly solid-like behavior for both hydrogels. Strain sweeps (0.1 – 100% shear strain) further confirmed this stability, as G’ remained greater than G’’ in the low-strain regime. Time-dependent rheological measurements showed rapid cross-linking of the precursor solutions, with G’ and G’’ stabilizing after an initial sharp increase. At approximately 800 s, the equilibrium G’ values of the QCS-PEG@TA and QCS-PEG hydrogels reached 1782.1 Pa and 1943.7 Pa, confirming sufficient structural integrity to maintain a stable three-dimensional network. These results demonstrated the hydrogels’ robust mechanical stability across a range of strains and frequencies, making them suitable for biomedical applications requiring consistent mechanical performance. Microstructural analysis *via* SEM revealed a porous, interconnected architecture that mimics the extracellular matrix (ECM) **(Figure 2E)**. This morphology supported cell adhesion and growth while facilitating efficient water retention and nutrient exchange, supporting both therapeutic and regenerative applications.

*In vitro* degradation profile of the hydrogels was evaluated to assess its stability and suitability for sustained therapeutic applications. The results showed that the hydrogel gradually degraded, retaining 50% of its original mass after 24 days of incubation **(Figure 2F)**. This controlled degradation rate highlighted the hydrogel’s potential for applications requiring sustained structural integrity and prolonged therapeutic effect, aligning with the demands of long-term biomedical interventions. Drug release test revealed that the QCS-PEG@TA hydrogel exhibited a rapid release (22.26% ± 4.38%) within the initial three days, followed by a gradual release profile over the subsequent 18 days (Figure S2). The cumulative release reached 42.02% ± 5.02% at day 21. These results showed that the hydrogel could sustain the release of TA, which was mainly depended on hydrogel degradation.

Otherwise, the adhesive performance of the hydrogels was evaluated to determine their suitability for adhering to various substrates, including biological tissues. The results demonstrated that QCS-PEG@TA hydrogels exhibited a peel shear strength of 72.00 ± 34.39 kPa on esophageal tissue, comparatively higher than that of QCS-PEG hydrogels (57.33 ± 8.63 kPa) **(Figure 2G)**. Cyclic compression tests showed a stable mechanical response throughout the entire cycle, with a slight decrease in peak compressive stress of 1.26%, indicating excellent fatigue resistance (Figure S3). The actual image showed that the hydrogel was firmly attached to the esophagus and closely adhered to the esophagus **(Figure 2H)**. Esophageal flushing experiments showed that the hydrogel remained intact and firmly adhered to the defects after 5 cycles of esophageal flushing at a rate of 10 mL/s (10 s each cycle) (Figure S4A). Then, we evaluated the flexibility of the hydrogel adapting to esophageal peristalsis, which involves repeated tissue bending. The isolated esophageal segment containing the implanted hydrogel was manually bent 180° for 30 s, which was repeated five times. The hydrogel was tightly attached to the surface of the esophagus, and no crack or separation from the tissue was observed during the whole bending process (Figure S4B). These findings highlighted the hydrogels’ strong adhesive properties, particularly on esophageal tissue, enabling efficient localized drug delivery and providing anti-inflammatory effects critical for therapeutic applications. Furthermore, the swelling behavior of QCS-PEG@TA and QCS-PEG hydrogels was evaluated *in vitro* to investigate their capacity for water absorption and its implications for drug release. Fully cross-linked hydrogel cylinders (1 cm in diameter, 0.5 cm in thickness) were immersed in PBS at 37℃. At predetermined intervals, the hydrogels were removed, gently blotted to remove surface water, weighed, and returned to the PBS solution for continued observation. Periodic mass measurements revealed swelling ratios exceeding 280% for both formulations **(Figure 2I)**. This substantial water absorption facilitated sustained drug release into surrounding tissues, enhancing the hydrogel’s anti-inflammatory efficacy. These findings demonstrated the hydrogels’ potential for controlled drug delivery systems, where swelling-mediated drug diffusion was critical for therapeutic outcomes.


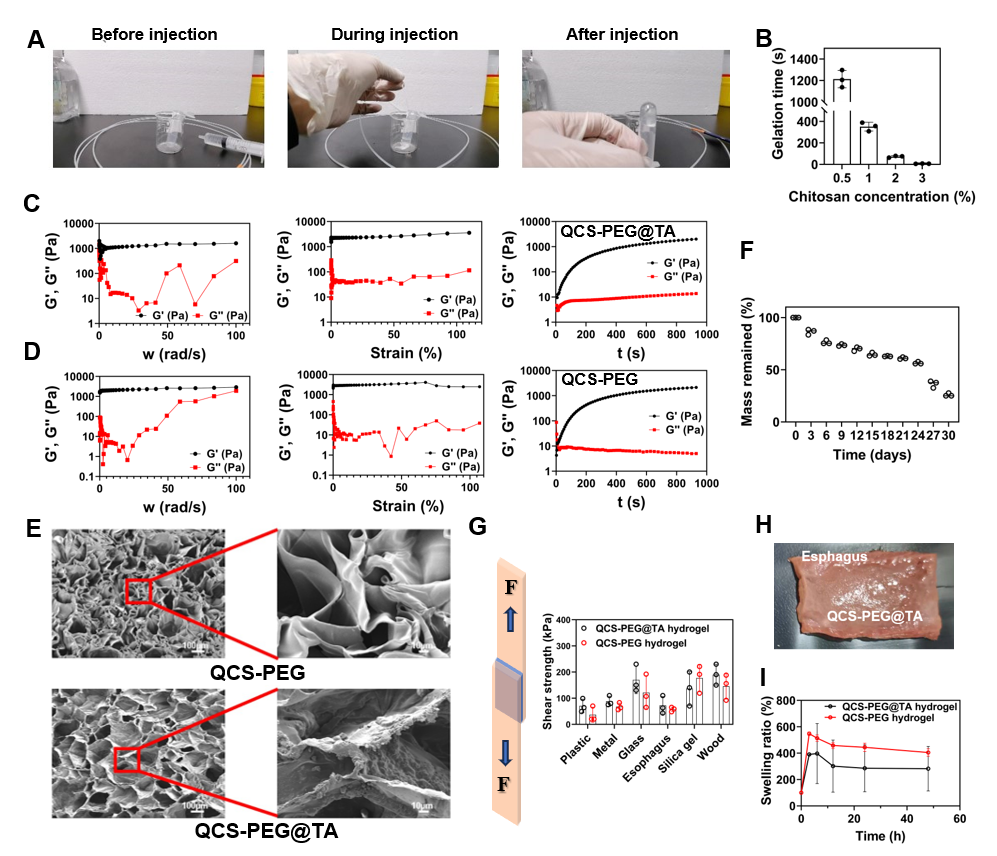


**Figure 2. Preparation and characterization of QCS-PEG@TA hydrogel.** (A) Hydrogel injection process through gastroscope catheter. (B) Gelation time of hydrogels (n = 3). (C) Rheological properties of QCS-PEG@TA hydrogel. (D) Rheological properties of QCS-PEG hydrogel. (E) Surface morphology of hydrogels. (F) Degradation curve of hydrogel with time (n = 3). (G) Peel shear strength of hydrogels on diverse materials (n = 3). (H) Actual image of hydrogel adhering to esophagus. (I) Swelling ratio of hydrogels (n = 3).

**Cytotoxicity,** **macrophage polarization and cell migration of hydrogel**

The cytocompatibility of QCS-PEG@TA and QCS-PEG hydrogels was evaluated using the CCK-8 assay. The results indicated that both hydrogels exhibited negligible cytotoxicity, with cell viability exceeding 90% in all test groups **(Figure 3A, B)**. These findings confirmed the excellent biocompatibility of the hydrogels, making them suitable for biomedical applications requiring direct interaction with cellular environments.

To evaluate immunomodulatory effects, BMDMs were cultured in the presence of QCS–PEG@TA. Flow cytometry analysis revealed a 30.8% increase in the M2 (anti-inflammatory) macrophage population following exposure **(Figure 3C, D)**. The levels of TNF-α and TGF-β in the cell supernatant were detected by ELISA method **(Figure 3E, F)**. The results indicated that QCS-PEG@TA significantly downregulated the level of TNF-α and significantly upregulate the level of TGF-β, consistent with established M2 polarization characteristics. This polarization effect underscores the gel’s potential to modulate immune responses, contributing to its therapeutic utility in inflammation-related conditions. In addition, excessive inflammatory response during ESD wound healing may lead to uncontrolled proliferation of submucosal fibrosis and contraction of scar tissue ultimately lead to esophageal stricture [1]. This process is mainly caused by the transformation of fibroblasts into myofibroblasts expressing α-smooth actin [35, 36]. Thus, the potential of the QCS-PEG@TA to inhibit fibroblast migration, a critical factor in the prevention of esophageal stricture, was assessed using *in vitro* migration assays. Fibroblast cells were cultured in the presence of the gel, and cell migration was monitored over time. The results revealed that the TA-carrying gel significantly inhibited fibroblast migration by 75.1% compared to controls **(Figure 3G, H)**. This inhibition was likely to reduce excessive tissue remodeling and fibrosis, effectively mitigating the risk of esophageal stricture. Furthermore, the scratch assays and cell proliferation assays using normal human esophageal squamous epithelial cells (HET-1A) were performed. A dose- and time-dependent effect of the QCS-PEG@TA hydrogel on cell migration (Figure S5A) was found. On day 1, higher concentration groups with the mass ratio of hydrogel to culture medium of 1:2.5 and 1:5 exhibited significantly reduced migration rates (26.62 ± 4.28% and 36.72 ± 5.97%, respectively) compared to the control (59.59 ± 10.69%), while lower concentrations (1:10 and 1:20) showed a mild inhibition (migration rate, 43.9 ± 6.52% and 42.97 ± 10.55%) (Figure S5B). However, the migration rates in the 1:10 and 1:20 groups increased to 65.02 ± 7.24% and 83.22 ± 3.22%, respectively, while the 1:20 group even exceeded that in the control group (82.24 ± 2.7%) on day 2. Consistent with these findings, higher concentrations (1:2.5 and 1:5) inhibited the proliferation of HET-1A cells on both day 1 and day 2, whereas lower concentrations (1:10 and 1:20) exhibited reduced inhibitory effects, with that of the 1:20 group approaching the control level by day 2. Furthermore, the proliferation of HET-1A cells in hydrogel was increased with the increase of dilution concentration (Figure S5C). These findings highlighted the gel’s capability to regulate cellular behavior, supporting its application in post-surgical interventions for esophageal conditions.


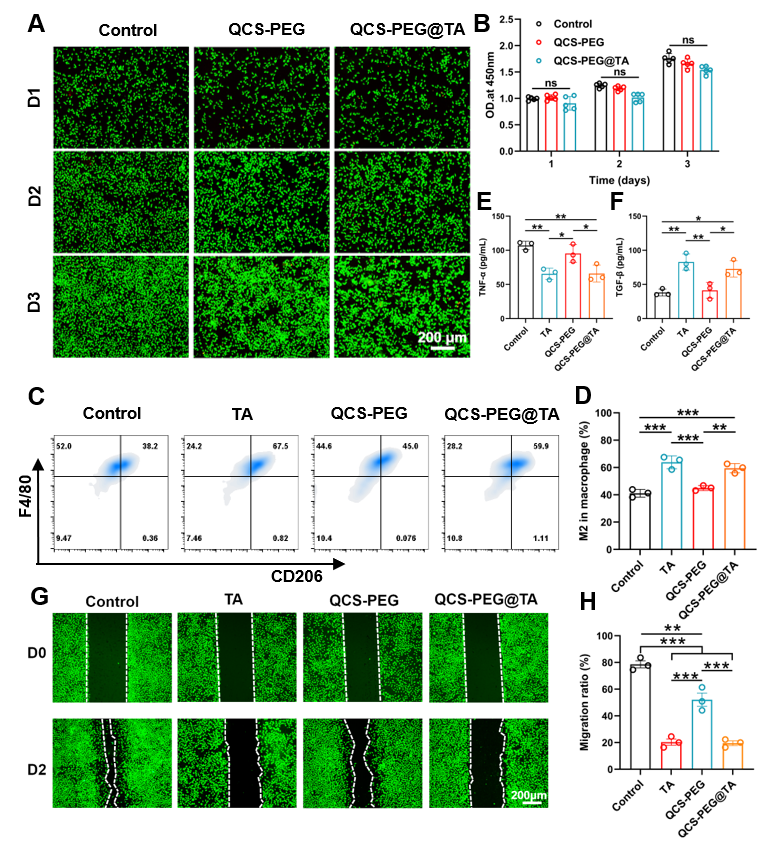


**Figure 3. Cytotoxicity,** **macrophage polarization and cell migration of hydrogel.** The cytotoxicity was evaluated by (A) live/dead staining on the 1st, 2nd, and 3rd day after processing with different materials and (B) CCK-8 assay (n = 5). (C) Flow cytometry analysis of F4/80 and CD206 expression. (D) Percentage of M2 macrophages (F4/80^+^CD206^+^, n = 3). (E) TNF-α, and F) TGF-β in cell supernatant after different treatments detected by ELISA (n = 3). (G) Cell migration assay and (H) migration ratio of L929 cells incubation with different treatments for 48 h (n = 3). **P* < 0.05, ***P* < 0.01 and ****P* < 0.001.

**Hydrogel prevention of esophageal stricture after ESD operation in animal model**

The efficacy of QCS-PEG@TA hydrogel in preventing post-ESD esophageal stricture was evaluated in a longitudinal porcine model **(Figure 4A)**. The animals were divided into four groups: untreated ESD model group (Control), local triamcinolone acetonide injection (TA), hydrogel-treated (QCS-PEG), and drug-loaded hydrogel-treated (QCS-PEG@TA), with three pigs per group. On day 0, the ESD model was established and corresponding treatment was carried out. In the QCS-PEG and QCS-PEG@TA groups, 8 mL of hydrogel was evenly sprayed onto the wound using a modified spray tube, while 1 mL (40 mg) of TA was injected at 4-6 submucosal sites in the TA group. The control group received no treatment. Afterwards, endoscopic esophageal examination was performed every seven days. Serial endoscopic evaluations were performed on days 7 and 28, followed by tissue harvesting for histological assessment **(Figure 4B)**.

Endoscopic evaluations revealed that the hydrogel-treated group exhibited superior outcomes compared to both the TA and control groups **(Figure 4C)**. The esophagus of the control group had approached near-total closure due to tissue hyperplasia and scar formation. The closure severity of the TA group and QCS-PEG group was milder than that of the control group. Sagittal gross specimen of the esophagus showed that the control group exhibited a clearly shortened and constricted esophagus with a rough, scarred mucosal surface, attributed to severe fibrotic contraction. In contrast, the QCS-PEG@TA group retained a nearly normal esophageal diameter, with a smooth, intact mucosa, which directly reflected the hydrogel’s ability to mitigate luminal narrowing (Figure S6). This is because TA hormone can reduce the level of inflammation at the wound, and QCS-PEG hydrogel can protect the wound from external factors. Meanwhile, high molecular weight chitosan has a certain degree of anti-inflammatory ability. Notably, the degree of esophageal closure in QCS-PEG@TA group was the lowest.

On postoperative day 7, the tissue at the wound site was biopsied and subjected to H&E and Masson’s staining, while the intact tissue at the wound site was removed on the 28th day. H&E results revealed that the infiltration of inflammatory cells in the QCS-PEG@TA group comparatively decreased, revealing the reduced inflammation at the wound site. The Masson’s staining results showed significant collagen deposition and severe tissue proliferation on days 7 and 28 **(Figure 4D, E)**. Quantitative histomorphometric analysis of Masson’s trichrome-stained sections demonstrated that the collagen deposition area in the biopsy tissue of the QCS-PEG@TA group (15.11 ± 2.3%) was significantly lower than that of the control group (37.73 ± 9.08%) **(Figure 4F)**. Full-thickness tissue samples harvested at day 28 permitted comprehensive assessment of healing progression. The collagen deposition in control groups reached 30.24 ± 6.63% area fraction by day 28. In contrast, QCS-PEG@TA treatment reduced collagen deposition to 14.56 ± 3.58% **(Figure 4G)**. These results emphasized the hydrogel’s advantages in wound protection and tissue regeneration through its localized delivery and sustained anti-inflammatory effects.


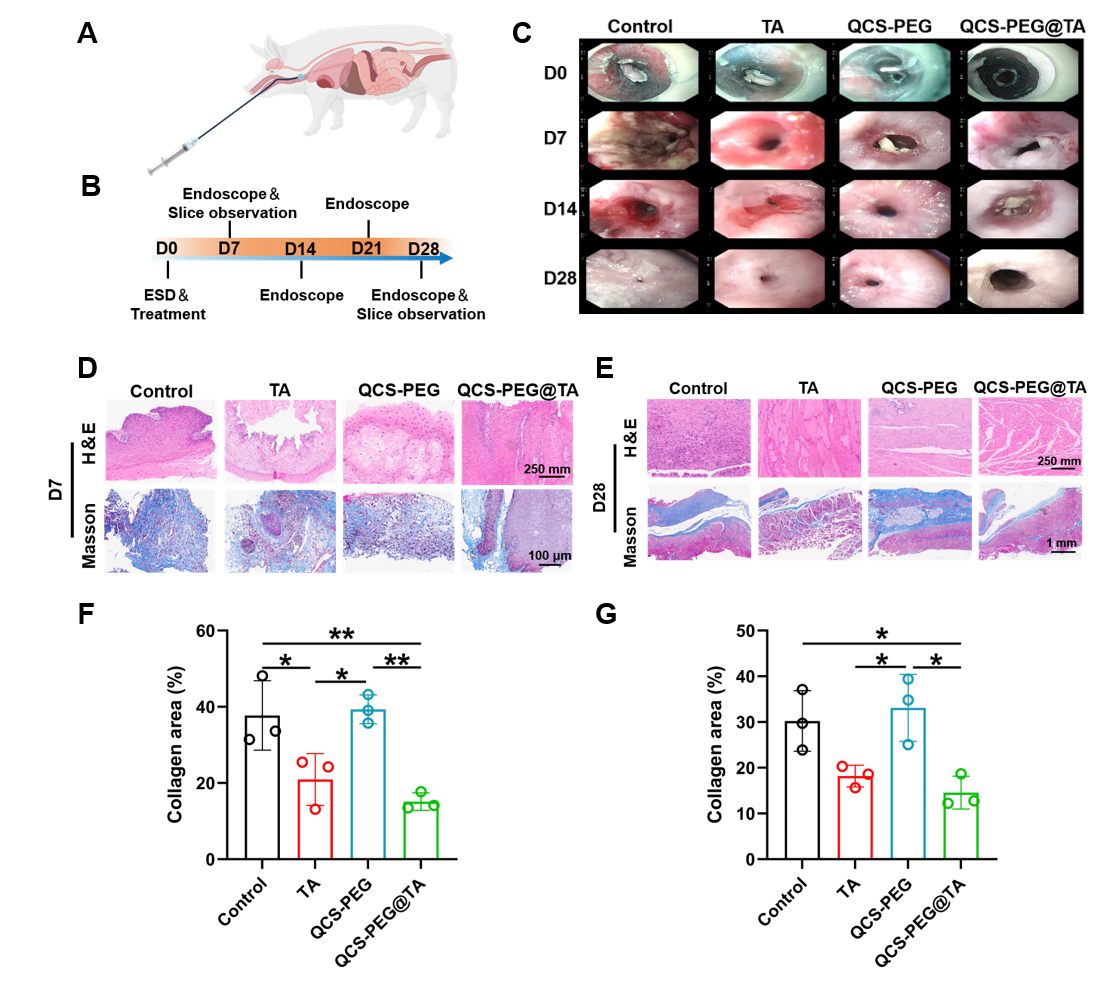


**Figure 4. QCS-PEG@TA hydrogel inhibited inflammation and fibrosis after ESD.** (A) Schematic diagram of hydrogel injection. (B) Schematic illustration of treatment in a ESD model. (C) Esophageal endoscopic assessment photos taken every seven days after ESD surgery. Representative images of H&E and Masson trichrome staining of the esophagus for each treatment on (D) day 7 and (E) day 28. Quantification of collage area on (F) 7th and (G) 28th day by Masson trichrome staining (n = 3). **P* < 0.05, ***P* < 0.01 and ****P* < 0.001.

Immunohistochemical analysis of esophageal tissues collected on day 7 post-ESD revealed distinct therapeutic modulation of inflammatory and fibrotic pathways **(Figure 5A)**. QCS-PEG@TA treatment significantly reduced the expression of pro-inflammatory cytokines. Quantification of mean optical density (MOD) indicated that TNF-α and TNF-β levels decreased by 55.0% and 41.2%, respectively, compared to controls (TNF-α: 0.09  ±  0.026 vs 0.2  ±  0.02; TNF-β: 0.1  ±  0.014 vs 0.17  ±  0.012) **(Figure 5B)**. These reductions confirmed the hydrogel’s capacity to suppress local inflammation during early wound healing.

Expression of markers associated with angiogenesis and fibroblast activation was significantly reduced following QCS-PEG@TA treatment. CD31, a marker of endothelial proliferation and neovascularization, showed a 45.0% reduction in MOD compared to the control group (MOD = 0.077  ±  0.012 vs 0.14  ±  0.02) **(Figure 5B)**. α-smooth muscle actin (α-SMA), indicative of fibroblast-to-myofibroblast transition, was also suppressed, with a 20.9% decrease in MOD relative to control (MOD = 0.072  ±  0.017 vs 0.09  ±  0.007). These results indicated that QCS-PEG@TA attenuated both vascular remodeling and fibrotic cell at the wound site.

The secretion of collagen caused by the activation of fibroblasts plays a central role in tissue repair and fibrosis [37]. Type I collagen deposition, quantified through immunohistochemistry, showed a 44.4% reduction in QCS-PEG@TA-treated wounds, consistent with Masson’s trichrome findings and confirming anti-fibrotic efficacy. Notably, CK5 expression increased by 1.4-fold, demonstrating enhanced basal epithelial cell regeneration critical for mucosal barrier restoration **(Figure 5B)**.

Sustained effects were observed at day 28. TNF-α and TNF-β levels in QCS-PEG@TA groups remained 41.79% and 38.03% below control values, respectively. Expression of CD31 and α-SMA was similarly suppressed, showing 39.5% and 40.3% reductions. Type I collagen continued to decline, reaching a 38% reduction compared to control, indicating long-term suppression of fibrotic remodeling. While CK5 expression exhibited no significance with controls, indicating completion of epithelial maturation into a stable stratified squamous architecture **(Figure 5C, D)**. These results collectively demonstrated that QCS-PEG@TA hydrogel provided both early and sustained modulation of key inflammatory and fibrotic pathways, supporting effective wound healing and stricture prevention following ESD.


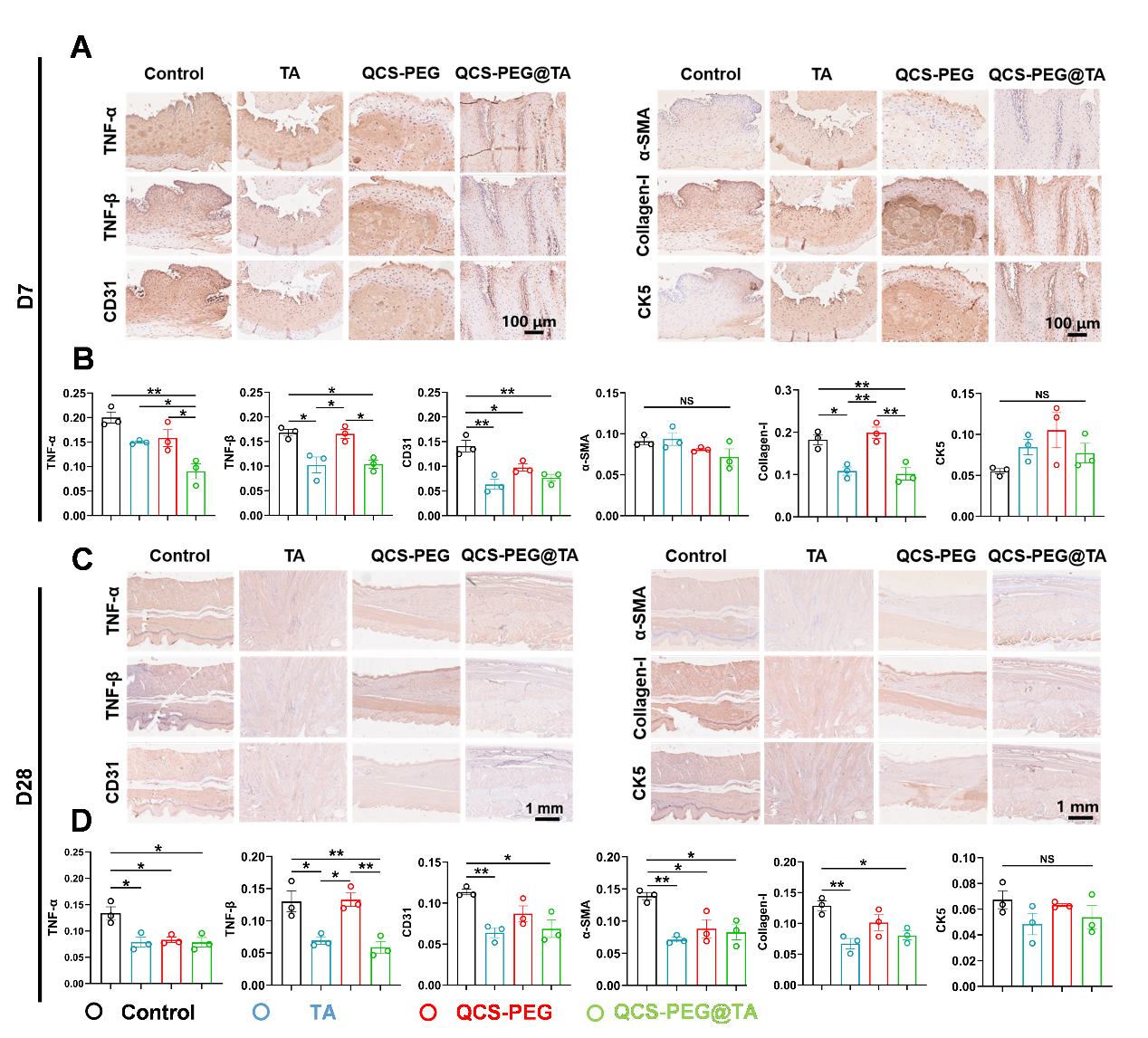


**Figure 5. QCS-PEG@TA hydrogel reduced the expression of inflammatory factors, angiogenesis and collagen deposition.** Immunohistochemistry staining (A) and quantitative statistics (B) of TNF-α, TNF-β, CD31, α-SMA, collagen-I, and CK5 on day 7 post-surgery (Scale bar, 100 μm; n = 3). Immunohistochemistry (C) and quantitative statistics (D) of TNF-α, TNF-β, CD31, α-SMA, collagen-I, and CK5 on day 28 post-surgery (Scale bar, 1 mm; n = 3). **P* < 0.05, ***P* < 0.01 and ****P* < 0.001.

**Discussion**

Esophageal stricture remains a critical post-operative complication of ESD, significantly compromising swallowing function and patient quality of life [18]. Despite numerous preventive strategies, including localized glucocorticoid injections, systemic anti-inflammatory therapies, and regenerative medicine approaches, a universally effective and standardized method remains elusive [21, 38]. To address this challenge, we engineered an effective injectable multifunctional hydrogel scaffold that integrated mechanical support, bio-adhesion, and drug delivery capabilities, specifically designed to prevent esophageal stricture.

The hydrogel system demonstrated distinct advantages, including enhanced adhesion to esophageal tissue, sufficient mechanical strength to resist contracture, non-toxicity, and rapid crosslinking, as validated in both *in vitro* and *in vivo* experiments. The adhesion of QCS-PEG hydrogel to the wound mainly depends on the electrostatic adsorption of the positive charge on QCS and the negative charge in the tissue, and the formation of hydrogen bond between PEG and the tissue surface [39]. The rapid crosslinking is clinically indispensable for ESD applications, where delayed gelation would allow premature hydrogel migration from mucosal defects. The quaternary ammonium groups on chitosan enhance water solubility at physiological pH to ensure the formation of hydrogel, instead of a precipitate. The three-dimensional porous network structure of the hydrogel facilitates localized delivery of triamcinolone acetonide, a guideline-recommended drug for esophageal stricture treatment [40]. The hydrogel’s ability to deliver TA locally avoids the systemic complications associated with oral or intravenous administration and mitigates the risk of esophageal perforation linked to traditional local injections. Furthermore, the United States Food and Drug Administration (FDA) has approved that chitosan and polyethylene glycol were GRAS (Generally Recognized as Safe) [41-43]. Also, a variety of antimicrobial dressings and drug vehicles using chitosan were approved by the FDA [44].

The immune microenvironment plays a critical role in esophageal stricture pathogenesis, with macrophages and fibroblasts acting as key mediators [45]. Multiple independent studies across different tissue contexts consistently demonstrate that TA intrinsically drives macrophage repolarization toward the M2 phenotype *in vivo* [46-48]. For example, in tendon injury models, TA-eluting hydrogels directly promoted macrophage M2 polarization, which orchestrated stem cell recruitment and tissue regeneration while mitigating fibrosis [49]. Our study demonstrated that hydrogel application effectively promoted macrophage polarization toward the anti-inflammatory M2 phenotype, resulting in down-regulation of inflammatory factors (TNF-α, TGF-β). The anti-fibrotic effect was complemented by the hydrogel’s ability to inhibit fibroblast migration as shown in *in vitro* experiments. These findings elucidate the underlying mechanisms of esophageal stricture and highlight the dual-action potential of the hydrogel in both modulating immune responses and attenuating fibrosis progression.

The animal model further validated the QCS-PEG@TA hydrogel’s efficacy, providing robust evidence of its anti-stricture effects, because TA in the hydrogel could widely act on the wound with the hydrogel, effectively preventing the formation of scar and reducing inflammation. Histopathological analysis revealed reduced inflammatory cells and lower levels of tissue fibrosis in the hydrogel-treated group compared to the control groups. At day 7, the seemingly counterintuitive reductions in CD31, α-SMA, and CK5 in the QCS-PEG@TA group compared to controls represent a deliberate and therapeutically favorable modulation of the wound microenvironment during the early inflammatory response. Specifically, the suppression of CD31 indicated the normalization of vasculature and prevention of aberrant angiogenesis, a key process in fibrosis. Concurrently, the reduction in α-SMA expression demonstrated the effective inhibition of fibroblast-to-myofibroblast transition, thereby curtailing the primary driver of collagen hypersecretion [50]. The diminished CK5 indicated restraint of reactive epithelial hyperplasia, a compensatory mechanism that often precedes stricture formation. It is crucial that early recalibration of the wound microenvironment does not impair regeneration, but strategically redirects healing towards physiological tissue recovery. The results clearly confirmed that collagen deposition was significantly reduced on day 28, and mucosal structure was fully restored at the endpoint.

In addition to its therapeutic effects, the hydrogel scaffold offers significant practical advantages. Its injectable nature allows for minimally invasive delivery, enabling precise lesion placement and reducing surgical risks. The mechanical and adhesive properties of the hydrogel facilitate its retention at the treatment site, overcoming the limitations of displacement or detachment observed with other biomaterials.

While this study provides a comprehensive evaluation of the hydrogel’s properties and efficacy, several challenges remain. Long-term studies are required to assess durability and safety over extended periods, particularly in clinical settings. Further optimization of the hydrogel’s composition may enhance its drug-loading capacity and mechanical performance. Moreover, integrating advanced therapeutic agents or leveraging multifunctional designs could expand its applicability to other fibrotic conditions.

Overall, this study presents an effective hydrogel-based intervention with significant potential for preventing esophageal stricture following ESD. By addressing both mechanical and biological aspects of tissue repair, this hydrogel provides a multifaceted solution to a persistent clinical challenge, paving the way for future advancements in minimally invasive and targeted therapies.

**Conclusions**

In this study, we developed an injectable QCS-PEG@TA hydrogel that addresses the clinical challenge of esophageal stricture following endoscopic submucosal dissection. The hydrogel demonstrated excellent injectability, strong tissue adhesion, and sustained structural integrity, allowing effective coverage of the resection site and localized delivery of anti-inflammatory agents. In a porcine ESD model, QCS-PEG@TA modulated immune responses, suppressed fibroblast activation, and reduced collagen deposition, resulting in significant attenuation of stricture formation. These findings position the hydrogel as a scalable and clinically translatable strategy for post-ESD wound management.

**Acknowledgements**

This work was funded by Beijing Tongzhou District Science and Technology Plan (KJ2024CX016), Capital’s Funds for Health Improvement and Research (2024-2-2025), Training Fund for Open Projects at Clinical Institutes and Departments of Capital Medical University (CCMU2024ZKYXY004) and Beijing Natural Science Foundation – Daxing Innovation Joint Fund (L146032).

**Conflict of Interest**

The authors declare no conflict of interest.

**Data Availability Statement**

The data that support the findings of this study are available from the corresponding author upon reasonable request.

**References**

1. Chen Z, Ding J, Wu C, Wei D, Sun J, Fan H, Guo Z. A review of hydrogels used in endoscopic submucosal dissection for intraoperative submucosal cushions and postoperative management. *Regen Biomater* **2023**;10:rbad064.

2. Pimentel-Nunes P, Dinis-Ribeiro M, Ponchon T, Repici A, Vieth M, De Ceglie A, Amato A, Berr F, Bhandari P, Bialek A, Conio M, Haringsma J, Langner C, Meisner S, Messmann H, Morino M, Neuhaus H, Piessevaux H, Rugge M, Saunders BP, Robaszkiewicz M, Seewald S, Kashin S, Dumonceau J-M, Hassan C, Deprez PH. Endoscopic submucosal dissection: European Society of Gastrointestinal Endoscopy (ESGE) Guideline. *Endoscopy* **2015**;47:829-54.

3. Yoshida M, Takizawa K, Nonaka S, Shichijo S, Suzuki S, Sato C, Komori H, Minagawa T, Oda I, Uedo N, Hirasawa K, Matsumoto K, Sumiyoshi T, Mori K, Gotoda T, Ono H, Yoshida M, Takizawa K, Ono H, Mori K, Nonaka S, Oda I, Shichijo S, Uedo N, Suzuki S, Kusano C, Gotoda T, Sato C, Hirasawa K, Komori H, Takeda T, Matsumoto K, Minagawa T, Fujii R, Sumiyoshi T, Yamasaki Y, Minashi K, Nakajima T, Kurokawa Y. Conventional versus traction-assisted endoscopic submucosal dissection for large esophageal cancers: a multicenter, randomized controlled trial (with video). *Gastrointest Endosc* **2020**;91:55-65.e2.

4. Jiang X, Liu Q, Fu M, Wang C, Zou R, Liu L, Wang M. Long-term outcomes of endoscopic submucosal dissection for early esophageal adenocarcinoma in the Eastern population: a comprehensive analysis. *J Gastrointest Surg* **2024**;28:1988-93.

5. Tang J, Kong F, Li J, Liu F, Kong X, Li Z. Independent risk factors for esophageal refractory stricture after extensive endoscopic submucosal dissection. *Surg Endosc* **2021**;35:3618-27.

6. Shibagaki K, Ishimura N, Oshima N, Mishiro T, Fukuba N, Tamagawa Y, Yamashita N, Mikami H, Izumi D, Taniguchi H, Sato S, Ishihara S, Kinoshita Y. Esophageal triamcinolone acetonide–filling method: a novel procedure to prevent stenosis after extensive esophageal endoscopic submucosal dissection (with videos). *Gastrointest Endosc* **2018**;87:380-89.

7. Yu M, Tan Y, Liu D. Strategies to prevent stricture after esophageal endoscopic submucosal dissection. *Ann Transl Med* **2019**;7(12):271.

8. Wen J, Yang Y, Liu Q, Yang J, Wang S, Wang X, Du H, Meng J, Wang H, Lu Z. Preventing Stricture Formation by Covered Esophageal Stent Placement After Endoscopic Submucosal Dissection for Early Esophageal Cancer. *Dig Dis Sci* **2014**;59:658-63.

9. Ono S, Fujishiro M, Niimi K, Goto O, Kodashima S, Yamamichi N, Omata M. Long-term outcomes of endoscopic submucosal dissection for superficial esophageal squamous cell neoplasms. *Gastrointest Endosc* **2009**;70:860-66.

10. Wen J, Lu Z, Linghu E, Yang Y, Yang J, Wang S, Yan B, Song J, Zhou X, Wang X, Meng K, Dou Y, Liu Q. Prevention of esophageal strictures after endoscopic submucosal dissection with the injection of botulinum toxin type A. *Gastrointest Endosc* **2016**;84:606-13.

11. Wynn, TA. Cellular and molecular mechanisms of fibrosis. *J Pathol* **2008**;214:199-210.

12. Wynn, TA, Ramalingam, TR. Mechanisms of fibrosis: therapeutic translation for fibrotic disease. *Nat Med* **2012**;18(7):1028-40.

13. Lupher ML, Jr., Gallatin WM. Regulation of fibrosis by the immune system. *Adv Immunol* **2006**;89:245-288.

14. Meng XM, Nikolic-Paterson DJ, Lan HY. TGF-beta: the master regulator of fibrosis. *Nat Rev Nephrol* **2016**;12:325-38.

15. Yang F, Hu Y, Shi Z, Liu M, Hu K, Ye G, Pang Q, Hou R, Tang K, Zhu Y. The occurrence and development mechanisms of esophageal stricture: state of the art review. *J Transl Med* **2024**;22:123.

16. Iwata K, Mikami Y, Kato M, Yahagi N, Kanai T. Pathogenesis and management of gastrointestinal inflammation and fibrosis: from inflammatory bowel diseases to endoscopic surgery. *Inflamm Regener* **2021**;41:21.

17. Wei Y, Qi M, Liu C, Li L. Astragalus polysaccharide attenuates bleomycin-induced pulmonary fibrosis by inhibiting TLR4/ NF-κB signaling pathway and regulating gut microbiota. *Eur J Pharmacol* **2023**;944:175594.

18. Yu J, Liu Y, Tao Y, Ruan R, Cui Z, Zhu S, Shi W. Prevention of Esophageal Stricture After Endoscopic Submucosal Dissection: A Systematic Review. *World J Surg* **2015**;39:2955-64.

19. Kinowaki S, Shimizu Y, Ono M, ZiJian Y, Tanaka I, Shimoda Y, Inoue M, Ishikawa M, Yamamoto K, Ono S, Ohnishi S, Sakamoto N. Experiment on endoscopic balloon dilation for esophageal stenosis after endoscopic submucosal dissection in pigs. *J Gastroenterol* **2021**;56:527-36.

20. Liu L, Qin J, Zeng C, Du R, Pan T, Ji J, Lu L, Chen L, Liu D, Yang J, He S, Zhu H, Teng G. Biodegradable PTX-PLGA-coated magnesium stent for benign esophageal stricture: An experimental study. *Acta Biomater* **2022**;146:495-505.

21. Hanaoka N, Ishihara R, Takeuchi Y, Uedo N, Higashino K, Ohta T, Kanzaki H, Hanafusa M, Nagai K, Matsui F, Iishi H, Tatsuta M, Ito Y. Intralesional steroid injection to prevent stricture after endoscopic submucosal dissection for esophageal cancer: a controlled prospective study. *Endoscopy* **2012**;44:1007-11.

22. Miyake M, Ishihara R, Matsuura N, Ueda T, Okubo Y, Kawakami Y, Tani Y, Yoshii S, Shichijo S, Kanesaka T, Yamamoto S, Takeuchi Y, Higashino K, Uedo N, Michida T, Matsunaga T. Predictors of stricture after non-circumferential endoscopic submucosal dissection of the esophagus and single-dose triamcinolone injection immediately after the procedure. *Gastrointest Endosc* **2023**;98:170-77.

23. Zhou X, Xu S, Ye L, Mao X, Chen Y, Wu J, Cai Y, Wang Y, Wang L, Li S. Progress of esophageal stricture prevention after endoscopic submucosal dissection by regenerative medicine and tissue engineering. *Regen Ther* **2021**;17:51-60.

24. Lai H, Yip H-C, Gong Y, Chan K-F, Leung KK-C, Chan MS, Xia X, Chiu PW-Y. MFGE8 in exosomes derived from mesenchymal stem cells prevents esophageal stricture after endoscopic submucosal dissection in pigs. *J Nanobiotechnology* **2024**;22:143.

25. Luc G, Charles G, Gronnier C, Cabau M, Kalisky C, Meulle M, Bareille R, Roques S, Couraud L, Rannou J, Bordenave L, Collet D, Durand M. Decellularized and matured esophageal scaffold for circumferential esophagus replacement: Proof of concept in a pig model. *Biomaterials* **2018**;175:1-18.

26. Li J, Chen G, Xu X, Abdou P, Jiang Q, Shi D, Gu Z. Advances of injectable hydrogel-based scaffolds for cartilage regeneration. *Regen Biomater* **2019**;6:129-40.

27. Song C, Liu R, Kong B, Gu Z, Chen G. Functional hydrogels for treatment of dental caries. *Biomed Technol* **2024**;5:73-81.

28. Xu J, Chang L, Xiong Y, Peng Q. Chitosan-Based Hydrogels as Antibacterial/Antioxidant/Anti-Inflammation Multifunctional Dressings for Chronic Wound Healing. *Adv Healthc Mater* **2024**;13:2401490.

29. Han Z, Bao L, Yu Y, Zhao Y, Wang M, Sun Y, Fu M, Zhou Q, Liu W, Cui W, Yu Y. Injectable short-fiber hydrogel with fatigue resistance and antibacterial properties for synergistic periodontitis therapy. *Chem Eng J* **2025**;520:166298.

30. Zhang Z, Wang J, Luo Y, Li C, Sun Y, Wang K, Deng G, Zhao L, Yuan C, Lu J, Chen Y, Wan J, Liu X. A pH-responsive ZC-QPP hydrogel for synergistic antibacterial and antioxidant treatment to enhance wound healing. *J Mater Chem B* **2023**;11:9300-10.

31. Di Y, Wang L, He W, Liu S, He Y, Liao J, Zhang R, Yin L, Xu Z, Li X. The utilization of chitosan/Bletilla striata hydrogels to elevate anti-adhesion, anti-inflammatory and pro-angiogenesis properties of polypropylene mesh in abdominal wall repair. *Regen Biomater* **2024**;11:rbae044.

32. Lin F, Li Y, Cui W. Injectable hydrogel microspheres in cartilage repair. *Biomed Technol* **2023**;1:18-29.

33. Zhang Z, Li H, Qian M, Zheng Y, Bao L, Cui W, Wang D. Up IGF-I via high-toughness adaptive hydrogels for remodeling growth plate of children. *Regen Biomater* **2025**;12:rbaf004.

34. Wang J, Liu X, Wang Y, Zhang Y, Gao R, Gao Y, Kong P, Huangfu Y, Zhang C, Feng Z, Huang P, Yang P, Wang W. Pro‐Regenerative Glycopeptide Hydrogel Activates Type 2 Immune Response for Wound Healing via Macrophage‐T Cell Crosstalk. *Adv Funct Mater* **2024**;34:2307711.

35. Yang F, Hu Y, Shi Z, Liu M, Hu K, Ye G, Pang Q, Hou R, Tang K, Zhu Y. The occurrence and development mechanisms of esophageal stricture: state of the art review. *J Transl Med* **2024**;22:123.

36. Di X, Li Y, Wei J, Li T, Liao B. Targeting Fibrosis: From Molecular Mechanisms to Advanced Therapies. *Adv Sci* **2025**;12:2410416.

37. Talbott HE, Mascharak S, Griffin M, Wan DC, Longaker MT. Wound healing, fibroblast heterogeneity, and fibrosis. *Cell Stem Cell* **2022**;29:1161-80.

38. Yamaguchi N, Isomoto H, Nakayama T, Hayashi T, Nishiyama H, Ohnita K, Takeshima F, Shikuwa S, Kohno S, Nakao K. Usefulness of oral prednisolone in the treatment of esophageal stricture after endoscopic submucosal dissection for superficial esophageal squamous cell carcinoma. *Gastrointest Endosc* **2011**;73:1115-21.

39. Rao P, Sun TL, Chen L, Takahashi R, Shinohara G, Guo H, King DR, Kurokawa T, Gong JP. Tough Hydrogels with Fast, Strong, and Reversible Underwater Adhesion Based on a Multiscale Design. *Adv Mater* **2018**;30:1801884.

40. Takeshita S, Zhao S, Malfait WJ, Koebel MM. Chemistry of Chitosan Aerogels: Three-Dimensional Pore Control for Tailored Applications. *Angew Chem Int Ed* **2021**;60:9828-51.

41. Kazemi Shariat Panahi H, Dehhaghi M, Amiri H, Guillemin GJ, Gupta VK, Rajaei A, Yang Y, Peng W, Pan J, Aghbashlo M, Tabatabaei M. Current and emerging applications of saccharide-modified chitosan: a critical review. *Biotechnol Adv* **2023**;66:108172.

42. Jiang M, Althomali RH, Ansari SA, Saleh EAM, Gupta J, Kambarov KD, Alsaab HO, Alwaily ER, Hussien BM, Mustafa YF, Narmani A, Farhood B. Advances in preparation, biomedical, and pharmaceutical applications of chitosan-based gold, silver, and magnetic nanoparticles: A review. *Int J Biol Macromol* **2023**;251:126390.

43. Suk JS, Xu Q, Kim N, Hanes J, Ensign LM. PEGylation as a strategy for improving nanoparticle-based drug and gene delivery. *Adv Drug Deliv Rev* **2016**;99:28-51.

44. Layek B, Rahman Nirzhor SS, Rathi S, Kandimalla KK, Wiedmann TS, Prabha S. Design, Development, and Characterization of Imiquimod-Loaded Chitosan Films for Topical Delivery. *AAPS PharmSciTech* **2019**;20:58.

45. Leng W, Li X, Dong L, Guo Z, Ji X, Cai T, Xu C, Zhu Z, Lin J. The Regenerative Microenvironment of the Tissue Engineering for Urethral Strictures. *Stem Cell Rev Rep* **2024**;20:672-87.

46. Siebelt M, Korthagen N, Wei W, Groen H, Bastiaansen-Jenniskens Y, Müller C, Waarsing J, de Jong M, Weinans H. Triamcinolone acetonide activates an anti-inflammatory and folate receptor–positive macrophage that prevents osteophytosis in vivo. *Arthritis Res Ther* **2015**;17:352.

47. Park J-Y, Kwon S, Kim S-H, Kang YJ, Khang D. Triamcinolone–Gold Nanoparticles Repolarize Synoviocytes and Macrophages in an Inflamed Synovium. *ACS Appl Mater Interfaces* **2020**;12:38936-49.

48. Li Y, Tu Q, Xie D, Chen S, Gao K, Xu X, Zhang Z, Mei X. Triamcinolone acetonide-loaded nanoparticles encapsulated by CD90(+) MCSs-derived microvesicles drive anti-inflammatory properties and promote cartilage regeneration after osteoarthritis. *J Nanobiotechnology* **2022**;20:150.

49. Freedman BR, Kuttler A, Beckmann N, Nam S, Kent D, Schuleit M, Ramazani F, Accart N, Rock A, Li J, Kurz M, Fisch A, Ullrich T, Hast MW, Tinguely Y, Weber E, Mooney DJ. Enhanced tendon healing by a tough hydrogel with an adhesive side and high drug-loading capacity. *Nat Biomed Eng* **2022**;6:1167-79.

50. Yang M, Li J, Gu P, Fan X. The application of nanoparticles in cancer immunotherapy: Targeting tumor microenvironment. *Bioact Mater* **2021**;6:1973-87.
